# A unified framework for correcting batch effects and integrating multi-omics data

**DOI:** 10.1038/s41598-026-42355-9

**Published:** 2026-03-05

**Authors:** Joung Min Choi, Heejoon Chae

**Affiliations:** 1 Department of Computer Science, Virginia Tech, Blacksburg, 24061 USA; 2 Division of Computer Science, Sookmyung Women’s University, Seoul, 04310 South Korea

**Keywords:** Cancer, Computational biology and bioinformatics

## Abstract

Multi-omics studies enable a comprehensive understanding of biological systems by integrating complementary molecular layers such as gene expression, DNA methylation, and chromatin accessibility. However, the generation of multi-omics data remains costly and labor-intensive, leading researchers to combine publicly available datasets collected from different cohorts, laboratories, and platforms. Integrating such heterogeneous datasets introduces substantial batch effects and technical variability that can obscure true biological structure. While numerous batch correction methods exist for single-omics data, systematic approaches for multi-omics batch effect correction remain limited. Correcting each omics layer independently risks disrupting cross-omics concordance and fails to ensure that samples are aligned within a unified multi-modal space, underscoring the need for coordinated, modality-aware harmonization that preserves shared molecular structure while removing technical variation across studies. To address this gap, we developed MoDAmix, a unified framework that leverages domain adaptation to remove technical variation while preserving shared molecular structure across omics layers. In particular, MoDAmix aligns feature distributions across batches and modalities through adversarial learning, enforcing consistency both within and between omics types to achieve coherent cross-omics integration. MoDAmix proceeds through four stages: (1) pre-training to learn initial feature representations, (2) adversarial adaptation to reduce batch effects within each omics type, (3) multi-omics adversarial alignment to harmonize modalities in a shared latent space, and (4) semi-supervised class alignment to refine subtype separability through pseudo-labeling and centroid consistency. Evaluations on both single-cell and bulk datasets–including mouse brain (gene expression and chromatin accessibility) and cancer cohorts (gene expression and DNA methylation)–demonstrated that MoDAmix effectively mitigates batch effects, improves clustering and classification performance, and preserves subtype structure across domains. Together, these results highlight MoDAmix as a robust framework for multi-omics batch effect correction and integration, enabling reliable cross-cohort analysis in systems biology and precision medicine. MoDAmix is publicly available at https://github.com/cbi-bioinfo/MoDAmix.

## Introduction

Advances in high-throughput sequencing technologies have enabled comprehensive profiling of multiple molecular layers, including the genome, epigenome, transcriptome, and proteome^1^. As a result, integrative multi-omics analysis has emerged as a powerful paradigm for characterizing complex biological systems, uncovering cross-layer regulatory relationships and coordinated molecular mechanisms that are often obscured in single-omics studies^2^. This integrative perspective is particularly important in heterogeneous diseases such as cancer, where multi-omics approaches have demonstrated substantial benefits across diverse applications^3^. In molecular subtyping, for example, combining multi-omics profiles with clinical information has improved patient stratification, enabling more accurate prognosis, enhanced interpretation of disease etiology, and personalized therapeutic strategies^4–6^. Likewise, in single-cell settings, jointly modeling complementary omics layers has led to more precise cell type identification and increased sensitivity in detecting subtle subpopulations, outperforming single-modality approaches^7,8^. Beyond classification tasks, integrative multi-omics has also accelerated biomarker discovery by capturing both genomic alterations and downstream functional dysregulation, supporting improved prediction of prognosis, treatment response, and drug sensitivity^9^. Taken together, by leveraging complementary molecular information, multi-omics integration enhances resolution, reproducibility, and biological interpretability, making it an essential task in contemporary biomedical research.

Despite its significant potential, the generation of multi-omics datasets remains costly, labor-intensive, and frequently limited by small sample sizes, posing a major practical barrier to widespread multi-omics research^2^. To overcome this limitation, researchers increasingly consolidate datasets from large public repositories such as TCGA^10^, ICGC^11^, GEO^12^, and ArrayExpress^13^ to expand cohort size, increase biological diversity, and enable more comprehensive modeling. However, integrating data produced across different laboratories, sequencing platforms, protocols, and time points inevitably introduces batch effects–systematic, non-biological variation arising from technical discrepancies in sample processing or measurement^14^. When left uncorrected, batch effects can overwhelm true biological signals, distort sample relationships, degrade clustering or classification performance, and ultimately lead to biased or misleading biological conclusions^15^. This problem is even more pronounced in multi-omics settings, where meaningful integration relies on preserving shared structure and coordinated patterns across molecular layers.

Although numerous batch correction tools exist for single-omics datasets, each is designed with modality-specific assumptions that limit their applicability to multi-omics integration. ComBat^16^ applies empirical Bayes shrinkage to adjust the mean and the variance by pooling information across multiple features for correcting batch-effects. Harmony^17^ applies an iterative anchoring strategy to align shared sample or cell identities across batches in a low-dimensional embedding space, which first combines the batches and projects the data into a dimensionally reduced space using PCA, and then uses an iterative procedure to remove the batch effects. Scanorama^18^ identifies mutual nearest neighbors across datasets to construct a unified representation from pairwise batch correspondences. While these approaches have shown strong performance in single-modality studies, they are primarily optimized for one omics layer at a time and do not fully address the added complexity of cross-omics batch variability.

Correcting each omics layer independently risks disrupting cross-omics concordance and fails to ensure that samples are aligned within a unified multi-modal space. As a result, existing batch correction tools remain insufficient for integrative multi-omics workflows, which require coordinated harmonization across modalities rather than isolated corrections. A dedicated, modality-aware harmonization strategy–one that preserves shared molecular structure while removing technical variation across studies–is therefore essential for reliable multi-omics integration and downstream discovery.

In this study, we present MoDAmix, a unified multi-omics batch correction framework that leverages domain adaptation to remove technical variation while preserving shared molecular structure across omics layers. MoDAmix transfers information learned from a source domain containing an adequately labeled multi-omics dataset to a different yet related target domain consisting of unlabeled multi-omics data collected from independent cohorts or experimental platforms. The proposed model proceeds through four sequential stages. In the first phase, modality-specific feature extractors and a shared classifier are pre-trained on labeled source data to learn initial low-dimensional representations. The second phase performs adversarial domain adaptation within each omics type, aligning source and target distributions to reduce batch effects at the single-omics level. In the third phase, the batch-corrected embeddings from all omics are integrated through a multi-omics feature extractor, where a shared adversarial alignment further harmonizes distributions across domains and modalities. In the final phase, semi-supervised class alignment refines the integrated latent space by combining supervised and pseudo-labeled learning, guided by a class-level centroid alignment to reinforce subtype consistency. We evaluated MoDAmix on diverse multi-omics tasks, including single-cell mouse brain data (gene expression and chromatin accessibility) for cell type identification and bulk cancer datasets (gene expression and DNA methylation) for leukemia and brain tumor subtyping. Performance was assessed through classification and clustering metrics, visualization of integrated feature spaces, and ablation analyses examining the contribution of each component. Comparisons with widely used batch correction tools show that MoDAmix more effectively mitigates batch effects and enhances class separability across datasets, facilitating the integration of heterogeneous multi-omics data through a unified and well-aligned feature space. MoDAmix is publicly available at https://github.com/cbi-bioinfo/MoDAmix.

## Results

### Performance evaluation on labeled dataset

For comprehensive evaluation of MoDAmix, we first assessed how its batch correction influences downstream predictive task using 5-fold cross-validation on labeled source datasets (see “Experimental design” section for details). MoDAmix was compared against widely used and recently developed batch correction methods, including Harmony^17^, ComBat^16^, and Scanorama^18^. To ensure a fair comparison, all methods were evaluated using the same preprocessed datasets and an identical classifier architecture; only the batch correction step differed among models. For each comparison method, batch correction was applied separately to each omics modality, and the resulting features were subsequently used for classification. The effectiveness of batch correction for the classification task was evaluated using accuracy and weighted F1-score metrics.

**Fig. 1:**
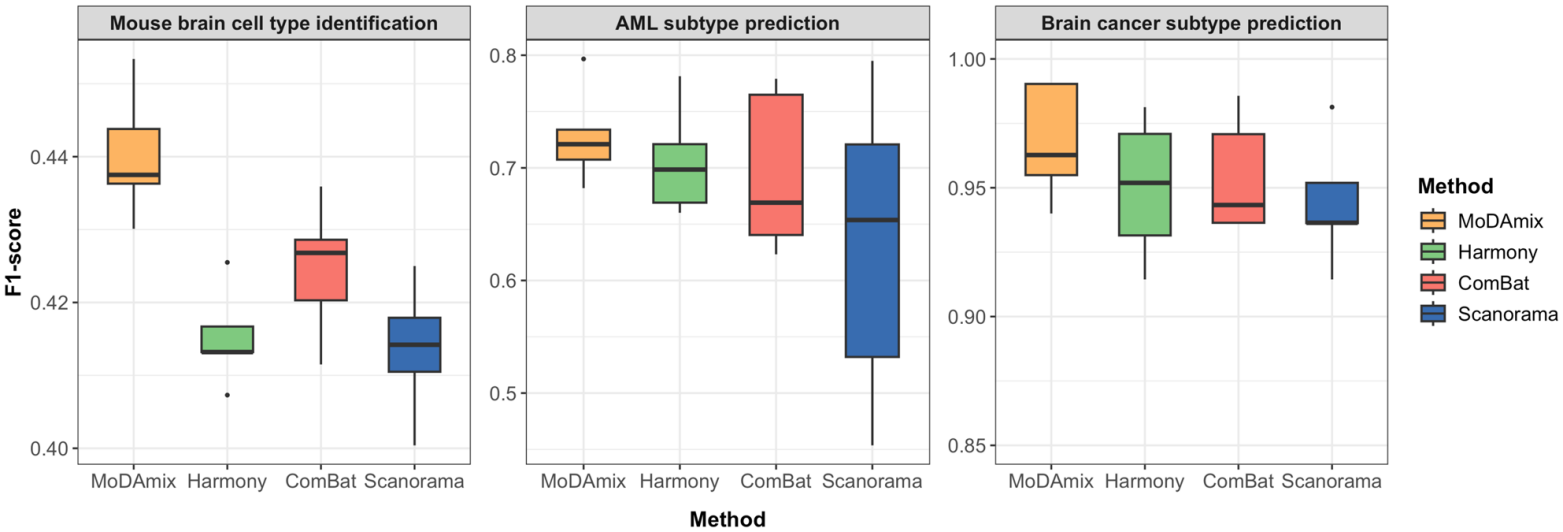
F1-score comparison of MoDAmix against widely-used batch correction methods, obtained from 5-fold cross-validation on labeled source datasets across benchmark tasks.

Across all three benchmark tasks, MoDAmix consistently outperformed existing batch correction methods (Fig. 1, Supplementary Material S1 and S2). From the results, MoDAmix achieved the highest performance across all datasets, demonstrating that effective elimination of technical variation through batch correction led to a more coherent and biologically consistent feature space. By reducing noise and aligning distributions across batches, MoDAmix preserved clearer class-discriminative structure, enabling the classifier to more accurately capture subtype-specific patterns. In the mouse brain cell type identification task, MoDAmix improved average classification accuracy from 0.413–0.423 in conventional methods to 0.448, reflecting more precise alignment between single-cell gene expression and chromatin accessibility features. In AML subtype classification, MoDAmix attained the average weighted F1-score of 0.728, surpassing Harmony (0.706), ComBat (0.695), and Scanorama (0.631). For brain cancer subtype prediction, MoDAmix achieved the highest overall classification performance (accuracy = 0.966, F1 = 0.967), confirming its robustness in preserving meaningful molecular signals across multi-omics layers. Collectively, these results highlight MoDAmix’s effectiveness in harmonizing data from heterogeneous sources while maintaining discriminative performance across both single-cell and bulk multi-omics applications.

### Comparison of prediction performance on target datasets using batch-corrected features

We next evaluated the performance of MoDAmix on target datasets, as one of the objectives is to accurately predict class labels for previously unseen samples lacking ground-truth annotations while mitigating batch effects through domain alignment. Assessing performance in this setting enables evaluation of whether batch correction successfully removes technical variation while preserving the intrinsic class-discriminative structure required for effective label transfer across domains. This experiment therefore examines the extent to which the learned representations maintain coherent class organization under domain shift.

In benchmark datasets where true labels were unavailable for the target data, we inferred class assignments using the model’s predictions and validated them through clustering-based performance metrics derived from the learned representations. Each predicted cell type or cancer subtype was treated as an individual cluster, and all experiments were repeated five times for robustness. We employed two standard clustering metrics: the Silhouette score, which measures how similar each sample is to its assigned cluster relative to others (higher values indicate better subtype separation), and the Davies–Bouldin Index (DBI), which assesses cluster compactness and separation (lower values indicate improved performance). MoDAmix generates multi-omics–integrated latent representations after batch correction and provides direct subtype predictions, whereas comparison methods output batch-corrected features for each single omics. To ensure a fair comparison, we applied K-means clustering to the batch-corrected features from Harmony, ComBat, and Scanorama, setting the number of clusters (*K*) equal to the number of known cell types or subtypes in each task. Likewise, for MoDAmix, we report both the results based on the model’s predicted labels (“MoDAmix (pred)”) and those obtained by applying K-means clustering directly to the integrated latent representations (“MoDAmix (k-means)”), using the same evaluation procedure as applied to the comparison methods.

Across all three benchmark tasks, MoDAmix consistently outperformed competing methods in both Silhouette score and DBI, demonstrating superior alignment and class-specific structure preservation in the integrated latent space (Tables 1 and 2). In the mouse brain cell type identification task, MoDAmix achieved the highest Silhouette scores (0.653 for predictions and 0.872 for K-means clustering) and the lowest DBI (1.482 and 0.471 for predictions and K-means clustering, respectively), substantially outperforming single-omics correction methods, which yielded Silhouette scores between –0.030 and 0.218 and DBI values above 1.4. In the AML subtype prediction task, MoDAmix again achieved superior clustering quality, with Silhouette scores of 0.981 for both prediction and K-means modes, and a remarkably low DBI of 0.139, confirming compact and well-separated subtype structures across cohorts. Similarly, for brain cancer subtype prediction, MoDAmix reached Silhouette scores of 0.966 and DBI of 0.128, both the best among all models, indicating stable subtype boundaries in the integrated representation. Collectively, these results demonstrate that MoDAmix effectively harmonizes multi-omics data while maintaining subtype separability and compactness, achieving robust alignment across heterogeneous domains.

**Table 1:** Average Silhouette score comparison demonstrating performance of MoDAmix and baseline methods on unlabeled target datasets.

Experiments	MoDAmix (pred)	MoDAmix (k-means)	Gene expression			DNA accessibility / DNA methylation		
			Harmony	ComBat	Scanorama	Harmony	ComBat	Scanorama
Mouse brain cell type identification	0.653	0.872	0.027	-0.030	0.057	0.117	-0.009	0.218
AML subtype prediction	0.981	0.981	0.136	0.079	0.138	0.143	0.054	0.197
Brain cancer subtype prediction	0.966	0.966	0.199	0.172	0.266	0.178	0.080	0.158

**Table 2:** Average DBI for predicting unlabeled target datasets based on the batch-corrected features from MoDAmix and baseline methods.

Experiments	MoDAmix (pred)	MoDAmix (k-means)	Gene expression			DNA accessibility / DNA methylation		
			Harmony	ComBat	Scanorama	Harmony	ComBat	Scanorama
Mouse brain cell type identification	1.482	0.471	4.548	9.908	3.604	2.253	4.876	1.448
AML subtype prediction	0.139	0.139	2.086	2.690	2.038	2.009	3.008	1.634
Brain cancer subtype prediction	0.128	0.128	1.710	1.851	1.545	1.951	2.792	1.870

### Performance evaluation on multi-omics integration performance

To directly assess each method’s ability to learn coherent multi-omics–integrated representations that characterize distinct cellular or patient populations, multi-omics latent embeddings were extracted from each target dataset using the competing methods, following the default or recommended settings provided in their original implementations. K-means clustering was applied to the learned representations from each model, and clustering performance was evaluated using the Silhouette score and Davies–Bouldin Index (DBI), with the number of clusters (K) set to the known number of cell types or subtypes for each task. For MoDAmix, K-means clustering was additionally applied directly to the integrated latent representations to enable a fair comparison. scMVAE was not included in the quantitative evaluation due to practical difficulties in reproducing the method, stemming from limited documentation and the absence of a complete, runnable tutorial in the publicly available implementation. As shown in Table 3, MoDAmix consistently outperformed competing multi-omics integration methods across all three benchmark tasks under both metrics. For example, in the adult mouse brain dataset, MoDAmix achieved the highest Silhouette score (0.872) and the lowest DBI (0.471), whereas MultiVI yielded the second-best performance with a Silhouette score of 0.537 and a DBI of 0.507. These results demonstrate that MoDAmix can effectively integrate multi-omics data into compact and well-separated latent representations, preserving biologically meaningful population structure.

**Table 3:** Comparison of clustering performance for multi-omics–integrated latent representations on target datasets using Silhouette score and Davies–Bouldin Index.

Experiments	Metric	MoDAmix	MOFA+	GLUE	MultiVI
Mouse brain cell type identification	Silhouette score	0.872	0.07	0.192	0.537
	DBI	0.471	1.709	1.419	0.507
AML subtype prediction	Silhouette score	0.981	0.167	0.049	0.257
	DBI	0.139	1.852	2.988	1.112
Brain cancer subtype prediction	Silhouette score	0.966	0.25	0.092	0.375
	DBI	0.128	1.704	2.535	1.048

### Exploring the effectiveness of batch effect correction based on UMAP visualization

**Fig. 2:**
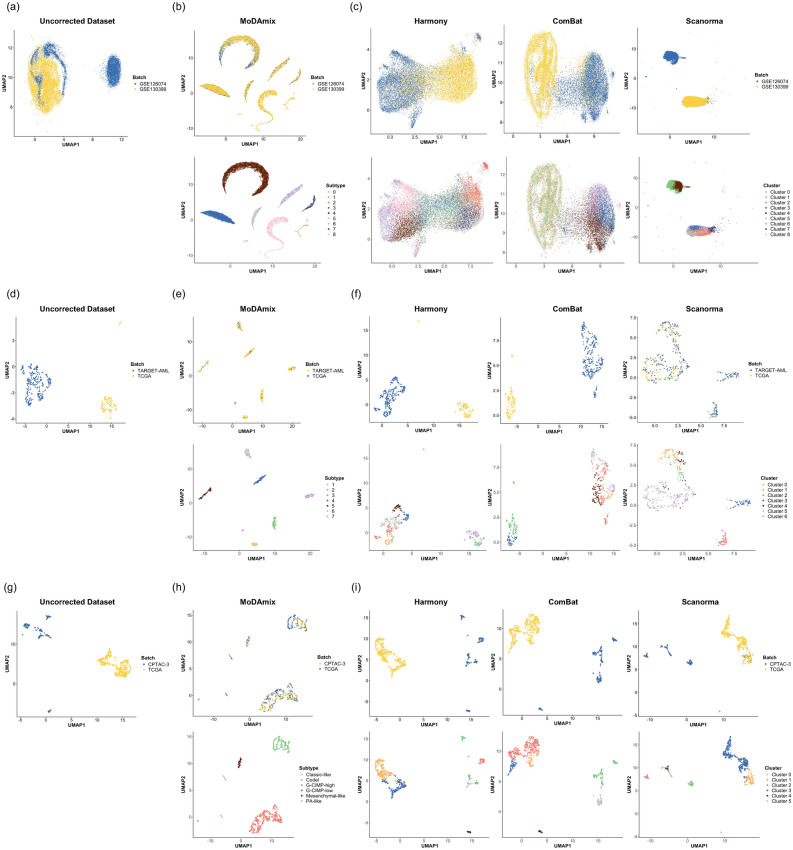
UMAP projections comparing uncorrected datasets and batch-corrected representations obtained from MoDAmix and other methods. For each dataset–(**a**–**c**) mouse brain, (**d–f**) acute myeloid leukemia (AML), and (g–i) brain cancer–the top panels show samples colored by batch, and the bottom panels show samples colored by predicted cell types or subtypes. (**a, d, g**) Uncorrected datasets before correction. (**b, e, h**) Multi-omics–integrated, batch-corrected features extracted using MoDAmix. (**c, f, i**) Batch-corrected representations from comparison methods, visualized based on gene expression features.

To investigate the effectiveness of batch effect correction, we projected the multi-omics–integrated, batch-corrected representations generated by MoDAmix into a two-dimensional space using Uniform Manifold Approximation and Projection (UMAP). The resulting embeddings were visualized by both predicted labels and batch identities to assess alignment quality and class separation. For comparison, we also visualized batch-corrected representations from Harmony, ComBat, and Scanorama, applying K-means clustering to their output features for annotation. UMAP projections were generated for gene expression data (Fig. 2) and for additional omics modalities such as DNA methylation or chromatin accessibility (Supplementary Material S3).

Across all datasets, MoDAmix achieved clear batch mixing and distinct class separation in the integrated latent space, while comparison methods exhibited either residual batch structure or cell type/subtype overlap. In the mouse brain dataset, MoDAmix effectively aligned cells from different batches (GSE126074 and GSE130399) while preserving cell type-specific topology, producing smooth transitions across cell populations. In contrast, Harmony and ComBat only partially mitigated batch effects, leaving visible domain-specific clusters, and Scanorama over-corrected, collapsing biological variation and fragmenting clusters. Similarly, in the AML and brain cancer tasks, MoDAmix integrated datasets from distinct cohorts (e.g., TCGA, TARGET, CPTAC-3) into cohesive manifolds where known subtypes were spatially well-separated and internally coherent, reflecting successful domain alignment and label consistency. These results demonstrate that MoDAmix corrects batch-specific variation in the omics-integrated space, while learning representations that preserve class boundaries across different datasets.

### Evaluating the contribution of adversarial alignment components

To evaluate the specific contribution of adversarial alignment to batch effect correction–the central mechanism underlying our framework–we performed an ablation analysis by removing the adversarial training component from both the single-omics and multi-omics stages of MoDAmix (denoted as ‘w/o AT for single, multi’) and re-evaluated its performance. The modified model was tested using 5-fold cross-validation on the source datasets and evaluated on the unlabeled target datasets using clustering metrics.

In MoDAmix (w/o AT for multi), the domain discriminator associated with the multi-omics alignment stage was removed, and the integrated latent representation was learned without joint adversarial supervision. All other components, including single-omics adversarial alignment, semi-supervised learning, and class-level centroid alignment, were retained unchanged. For MoDAmix (w/o AT for single, multi), adversarial training was removed entirely by disabling all domain discriminators at both the single-omics and multi-omics stages. In this setting, the model relies solely on supervised learning on labeled source data and semi-supervised learning on target data, without any explicit domain adversarial objectives.

As summarized in Tables 4 and  5, eliminating adversarial alignment led to consistent declines in both classification and clustering performance. On the labeled source data, accuracy and F1-score decreased across all tasks, indicating that adversarial learning improves the model’s ability to capture class-discriminative structure. On the target datasets, Silhouette scores were markedly reduced (e.g., from 0.999 to 0.170 for the mouse brain dataset), while DBI values increased, reflecting diminished cluster compactness and cross-domain coherence. These results suggest that adversarial alignment plays a key role in promoting domain-invariant representation learning and in maintaining subtype consistency across heterogeneous datasets.

We further investigated whether multi-omics adversarial alignment specifically contributes to improved integration in the joint latent space. To this end, we constructed a variant of the model, MoDAmix (w/o AT for multi), in which the adversarial alignment at the multi-omics stage was removed and the modality-specific representations were simply concatenated before the final semi-supervised and class alignment phases. As shown in Tables 4 and  5, this variant yielded lower overall performance compared to the full MoDAmix framework. On the labeled source datasets, the classification accuracy and F1-scores of MoDAmix (w/o AT for multi) were slightly reduced, particularly for the AML task (accuracy 0.708 vs. 0.735; F1-score 0.689 vs. 0.725). The differences became more evident on the unlabeled target datasets, where the Silhouette scores dropped substantially (e.g., 0.978 $\rightarrow$ 0.776 for AML, 0.981 $\rightarrow$ 0.883 for brain cancer), and DBI values increased, indicating less compact and more overlapping clusters. These findings highlight that adversarial alignment in the joint multi-omics latent space contributes meaningfully to harmonizing modality-specific information and preserving coherent subtype structures after integration.

**Table 4:** Average classification performance comparing MoDAmix and its variant removing batch effect correction stages, using 5-fold cross-validation on source datasets.

Experiment	MoDAmix		MoDAmix (w/o AT for multi)		MoDAmix (w/o AT for single, multi)	
	Accuracy	F1-score	Accuracy	F1-score	Accuracy	F1-score
Mouse brain cell type identification	0.448	0.440	0.449	0.410	0.417	0.420
AML subtype prediction	0.735	0.725	0.708	0.689	0.701	0.703
Brain cancer subtype prediction	0.966	0.967	0.966	0.967	0.964	0.960

**Table 5:** Clustering performance on unlabeled target datasets comparing MoDAmix and its variant removing batch effect correction stages.

Experiment	MoDAmix		MoDAmix (w/o AT for multi)		MoDAmix (w/o AT for single, multi)	
	Silhouette score	DBI	Silhouette score	DBI	Silhouette score	DBI
Mouse brain cell type identification	0.999	0.457	0.918	0.351	0.170	1.582
AML subtype prediction	0.978	0.030	0.776	0.306	0.028	1.566
Brain cancer subtype prediction	0.981	0.049	0.883	0.138	0.255	0.814

## Discussion and conclusions

In this study, we developed MoDAmix, a unified domain adaptation framework for batch correction and integration of multi-omics data. Through a staged alignment strategy, MoDAmix effectively harmonizes heterogeneous datasets while preserving meaningful molecular variation across omics layers. Systematic evaluations on both single-cell and bulk multi-omics benchmarks demonstrated that MoDAmix consistently outperformed widely used batch correction tools such as Harmony, ComBat, and Scanorama in classification, clustering, and visualization analyses. The framework achieved improved alignment between the source and target domains, reduced residual batch structure, and maintained clearer subtype or cell-type separability in the integrated latent space.

The results highlight the importance of combining adversarial learning and semi-supervised optimization for robust cross-domain representation. Adversarial alignment at both single-omics and multi-omics levels was shown to substantially improve feature consistency and cluster compactness, while the class-level centroid alignment further refined subtype boundaries across domains. The ablation analyses confirmed that removing adversarial components led to a measurable decline in both classification and clustering performance, underscoring their contribution to domain-invariant feature learning. These findings collectively suggest that batch correction in multi-omics analysis benefits from coordinated alignment across modalities rather than independent, modality-specific corrections.

Beyond its methodological improvements, MoDAmix demonstrates practical utility for real-world integrative studies. In the single-cell mouse brain dataset, the framework successfully aligned transcriptomic and chromatin accessibility profiles to produce coherent cell-type manifolds. In the bulk cancer datasets, it effectively harmonized RNA-seq and DNA methylation data collected across different cohorts and experimental platforms, leading to stable and well-separated molecular subtypes. These outcomes indicate that MoDAmix can serve as a generalizable solution for large-scale multi-omics integration where technical variability and heterogeneous data sources are major obstacles.

In practical multi-omics integration settings, datasets often exhibit weak cross-modality correlation and substantial heterogeneity arising from differences in experimental protocols and data generation processes. MoDAmix is designed to operate under these conditions and does not rely on strong assumptions of direct correspondence between omics modalities. Instead, it emphasizes reducing domain-induced variation through adversarial alignment at both the single-omics and integrated multi-omics stages, while semi-supervised class alignment promotes consistency of class structure across domains. This design supports stable representation learning and preservation of class-discriminative structure in heterogeneous multi-omics datasets.

While MoDAmix achieves promising results, several avenues remain for further improvement. Future work could extend the model to accommodate additional omics layers, such as proteomics or metabolomics, and explore attention-based or graph-based architectures to better capture cross-omic dependencies. Incorporating biological priors–such as gene regulatory networks or pathway information–may also enhance interpretability and robustness. Moreover, optimizing computational efficiency and scalability will be crucial for applying MoDAmix to ever-growing multi-omics consortia and single-cell atlases.

In conclusion, MoDAmix provides a principled and extensible framework for correcting batch effects and integrating multi-omics data. By unifying adversarial and semi-supervised learning across both single- and multi-omics spaces, it enables the generation of well-aligned, biologically consistent representations suitable for downstream analyses such as subtyping, biomarker discovery, and patient stratification. As multi-omics data continue to expand in scale and complexity, frameworks like MoDAmix will play an essential role in advancing reproducible and integrative systems biology.

## Methods

### Overview

MoDAmix is a unified framework designed to correct batch effects in multi-omics datasets through staged alignment in both modality-specific and shared latent spaces. The model takes source and target dataset as input, where the source dataset refers to a multi-omics dataset with labeled annotation, while the target dataset refers to a different yet related unlabeled multi-omics dataset collected from independent cohorts or experimental platforms. Let $X^{(o)}_s \in \mathbb {R}^{n_s \times m^{(o)}}$ and $X^{(o)}_t \in \mathbb {R}^{n_t \times m^{(o)}}$ denote the source (labeled) and target (unlabeled) data for omics type $o \in \{1, \dots , O\}$, where $m^{(o)}$ is the number of features for modality *o* and $n_s$ and $n_t$ are the number of samples in source and target data, respectively. Each source sample is associated with a class label from *K* categories. The MoDAmix workflow consists of four major stages: (1) pre-training feature extractors and classifier, (2) single-omics domain adaptation, (3) multi-omics adversarial alignment, and (4) semi-supervised class alignment.

**Fig. 3:**
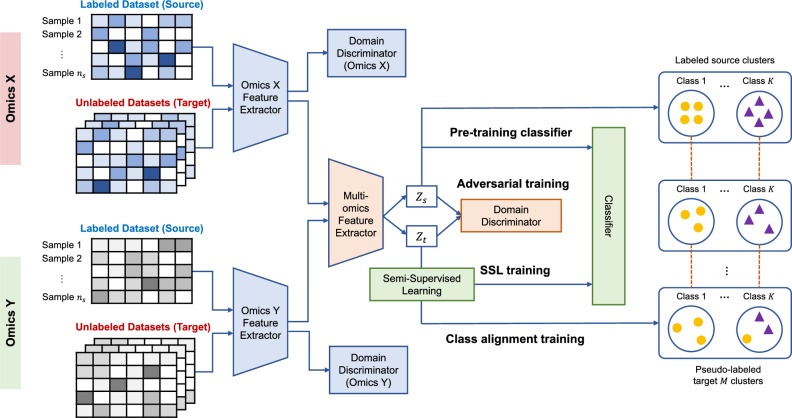
Illustration of the MoDAmix model for multi-omics batch effect correction.

MoDAmix first learns initial low-dimensional representations by pre-training feature extractors and classifier using labeled source data. Then, single-omics domain adaptation is performed, which refers to adversarial training performed independently within each omics modality, where a modality-specific domain discriminator is used to align source and target feature distributions for that modality alone. This stage aims to remove batch effects that are intrinsic to each omics type before cross-modal integration. After that, multi-omics adversarial alignment is applied after the modality-specific embeddings have been batch-corrected and concatenated. In this stage, a shared domain discriminator operates on the integrated multi-omics representation to further align source and target samples in a joint latent space, thereby mitigating residual cross-domain and cross-modality discrepancies that are not addressed by single-omics correction alone. In the final phase, semi-supervised class alignment refines the integrated latent space by combining supervised and pseudo-labeled learning, guided by a class-level centroid alignment to reinforce subtype consistency. The overall workflow is shown in Fig. 3.

### Phase 1: pre-training feature extractors and classifier

In the first phase, MoDAmix learns initial representations by training omics-specific feature extractors $F^{(o)}(\cdot )$ and a shared multi-omics feature extractor $G(\cdot )$. For each omics modality, source samples are passed through $F^{(o)}$, and the resulting latent vectors are used to train a classifier $C(\cdot )$ having softmax function as the final layer, on the labeled source domain. The objective is to minimize the cross-entropy loss :

1 $$\begin{aligned} \mathscr {L}_{\text {PT}}^{(o)} = -\frac{1}{n_s}\sum _{i=1}^{n_s}\sum _{k=1}^{K} y_{i,k}\log \big (C(F^{(o)}(x^{(o)}_{s,i}))\big ), \end{aligned}$$

where $y_{i,k}$ denotes the binary indicator if the class label *k* is the correct label for sample *i*. After optimizing each $F^{(o)}$, the modality-specific embeddings are concatenated and fed into $G(\cdot )$ to learn an initial shared latent representation. The shared extractor is trained with the same supervised objective:

2 $$\begin{aligned} \mathscr {L}_{\text {shared-PT}} = -\frac{1}{n_s}\sum _{i=1}^{n_s}\sum _{k=1}^K y_{i,k}\log \big (C(G(z_i))\big ), \end{aligned}$$

where $z_i$ is the concatenated embedding of sample *i* across omics layers.

### Phase 2: single-omics domain adaptation for initial batch effect correction

Once pre-training is complete, MoDAmix performs adversarial domain adaptation within each omics modality to correct batch effects at the single-omics level. For each modality *o*, a domain discriminator $D^{(o)}(\cdot )$ is trained to distinguish whether an embedding originates from the source or target dataset, while $F^{(o)}$ is optimized to produce domain-invariant representations. In this phase, the feature extractor and the domain discriminator are engaged in a competitive learning process. The adversarial training objective is formulated as:

3 $$\begin{aligned} \min _{D^{(o)}}\max _{F^{(o)}} \mathscr {L}_{\text {DA}}^{(o)} = -\frac{1}{n_s+n_t}\sum _{i=1}^{n_s+n_t}\sum _{j=1} d_{i,j} \log \big (D^{(o)}(F^{(o)}(x^{(o)}_i))\big ), \end{aligned}$$

where $d_{i,j}$ is the domain label of sample *i*. This step reduces batch effects independently in each omics layer before cross-modal integration.

### Phase 3: multi-omics adversarial alignment for batch effect correction and integration

To harmonize feature distributions across datasets and integrate multiple omics into a shared latent space, MoDAmix performs a second stage of adversarial alignment at the multi-omics level. The batch-corrected representations from each omics–obtained after single-omics adaptation–are concatenated and passed to a multi-omics feature extractor $G(\cdot )$, which learns a unified latent representation across modalities. A shared domain discriminator $D_{\text {shared}}(\cdot )$ is jointly trained to classify the domain origin of each sample, while $G(\cdot )$ is optimized to generate domain-invariant multi-omics embeddings by deceiving the discriminator. The corresponding adversarial objective is defined as :

4 $$\begin{aligned} \min _{D_{\text {shared}}}\max _{G} \mathscr {L}_{\text {shared-DA}} = -\frac{1}{n_s+n_t}\sum _{i=1}^{n_s+n_t}\sum _{j=1} d_{i,j} \log \big (D_{\text {shared}}(G(z_i))\big ), \end{aligned}$$

where $d_{i,j}$ denotes the domain label of sample *i*, and $z_i$ represents the concatenated feature vector from all modalities. Through this phase, MoDAmix aligns the global structure of the integrated representations across source and target domains, reducing residual cross-omics discrepancies that remain after single-omics correction. The resulting joint latent space serves as a batch-invariant and modality-consistent embedding, forming the foundation for semi-supervised class alignment and class prediction in the final phase.

### Phase 4: semi-supervised class alignment

In the final stage, MoDAmix refines the integrated latent space through semi-supervised class alignment, which jointly utilizes labeled source samples and pseudo-labeled target samples. Pseudo-labels for target data are assigned based on the classifier’s confidence scores by selecting the class with the highest predicted posterior probability. This iterative process allows the model to progressively incorporate target samples into training, enhancing representation consistency between the source and target domains. The objective combines supervised learning on the source data with pseudo-labeled learning on the target data as :

5 $$\begin{aligned} \mathscr {L}_{SSL}&= -\frac{1}{n_s}\sum _{i=1}^{n_s}\sum _{j=1}^{K}y_{i,j}\log ({p}_{i,j}) - \alpha (t)\frac{1}{n_t}\sum _{i=1}^{n_t}\sum _{j=1}^{K} {y}_{i,j}'\log ({p}_{i,j}), \end{aligned}$$

where $y_{i,j}$ and ${y}_{i,j}'$ denote the source class labels and pseudo-labels for target samples, respectively, and $p_{i,j}$ refers to the predicted posterior probability that sample *i* belongs to class *j*, as produced by the softmax layer of the classifier. The balancing term $\alpha (t)$ controls the relative influence of the unlabeled data. It is scheduled to gradually increase during training, stabilizing optimization by preventing premature reliance on uncertain pseudo-labels^19,20^:

6 $$\begin{aligned} \alpha (t)= {\left\{ \begin{array}{ll} 0, & t<T_{1},\\ \frac{t-T_{1}}{T_{2}-T_{1}}\alpha _{f}, & T_{1}\le t<T_{2},\\ \alpha _{f}, & T_{2}\le t, \end{array}\right. } \end{aligned}$$

with *t* denoting the epoch number, thresholds $T_{1}=100$ and $T_{2}=200$ and $\alpha _f = 0.01$.

To further align decision boundaries across domains, MoDAmix applies a class-level centroid alignment that encourages samples from the same class to cluster around a shared centroid, regardless of domain origin. For each class *k* and domain *m*, global and domain-specific centroids ($C^{k}$ and $C^{k}_{m}$) are computed in the shared latent space, and their distance is minimized as

7 $$\begin{aligned} \mathscr {L}_{CA} = \frac{1}{K}\sum _{k=1}^{K}\sum _{m=1}^{M}\Vert C^{k} - C^{k}_{m}\Vert _2^2. \end{aligned}$$

This alignment term reinforces inter-domain consistency while preserving class separability. Through this process, MoDAmix refines domain-invariant features into a compact and well-structured latent representation, ensuring consistent subtype discrimination across heterogeneous multi-omics datasets.

### Hyperparameter setting

Each module in MoDAmix was implemented as a fully connected neural network comprising two hidden layers. The single-omics feature extractor consisted of 1,024 and 512 hidden nodes, while the multi-omics feature extractor included 512 and 256 nodes. The classifier contained two layers with 128 and 64 hidden nodes, followed by a softmax layer. For both the single-omics and multi-omics batch correction stages, the domain discriminators were designed with two layers of 256 and 64 nodes. All layers employed the LeakyReLU activation function, and batch normalization was applied after each hidden layer to stabilize training. Model training employed the Adam optimizer ^21^, with learning rates set to $10^{-4}$ for the feature extractors, $10^{-5}$ for the classifier, and $10^{-6}$ for all domain discriminators. The models were trained for 500 epochs in all stages, except for the final fine-tuning phase, which was iteratively optimized for 800 epochs. The balancing parameter in the semi-supervised loss, $\alpha _f$, was set to 0.01. The MoDAmix framework was implemented using the PyTorch library (version 2.8.0).

### Experimental design

To comprehensively evaluate MoDAmix across diverse batch correction scenarios, we designed three analysis tasks: (1) adult mouse brain cell type identification, (2) acute myeloid leukemia (AML) subtype classification, and (3) brain cancer subtype prediction. For each task, we collected a source dataset with known annotations and an unlabeled target dataset generated from distinct cohorts or experimental platforms to simulate realistic cross-domain variation. The source and target datasets were obtained from independent studies, each dataset was treated as a separate batch, resulting in two batches per task (one source batch and one target batch), with batch sizes corresponding to the number of samples in each dataset. A detailed summary of all datasets is provided in Table 6.

#### Single-cell multi-omics task (adult mouse brain)

For the adult mouse brain task, we employed a single-cell multi-omics dataset comprising single-cell gene expression and chromatin accessibility profiles. The goal was to assess MoDAmix’s ability to align transcriptomic and epigenomic modalities for accurate cell type identification across batches. Preprocessing followed established single-cell multi-omics workflow^8^. Genes expressed in fewer than 30 cells were removed, and read counts were normalized by library size followed by log transformation. We then selected 3,000 highly variable genes (HVGs) using the Scanpy Python package^22^. For chromatin accessibility, we summarized accessibility peaks within each gene body region to infer gene activity scores associated with transcription^23^. These scores were aggregated for the same set of 3,000 HVGs to ensure feature correspondence between the two modalities.

#### Bulk multi-omics tasks (AML and brain cancer)

For the AML and brain cancer subtype prediction tasks, we collected RNA-seq–based gene expression and DNA methylation datasets from multiple public cohorts. Methylation profiles were generated using the Illumina Human Infinium 27K, 450K, and 850K platforms. Data preprocessing followed the workflows established in previous studies^24,25^. CpG sites common to both source and target datasets were retained. CpGs with more than 20% missing values were excluded, and the remaining missing values were imputed using the median. For feature selection, CpGs were grouped via K-means clustering, and the median beta value within each cluster was computed, yielding 3,000 CpG clusters. For gene expression preprocessing, lowly expressed genes were filtered, normalized, and log-transformed as described above. Genes were then matched to the retained CpG features based on promoter–CpG associations to ensure cross-omics consistency. The resulting expression feature set consisted of 3000 matched HVGs aligned with the methylation features.

**Table 6:** Datasets used for MoDAmix evaluation.

Benchmark Task	Adult mouse brain cell type identification		AML subtype prediction		Brain cancer subtype prediction	
Dataset	GSE130399^26^	GSE126074^27^	TCGA-LAML^10^	TARGET-AML^28^	TCGA-LGG^10^	CPTAC-3^29^
Number of samples	11366	10309	147	335	531	275
Sequencing protocol	Paired-seq	SNARE-seq	RNA-seq, Illumina 27K and 450K		RNA-seq, Illumina 450K and 850K	
With annotation	9 cell types	-	7 subtypes	-	6 subtypes	-

### Comparison with other batch correction and multi-omics integration methods

We first compared the performance of MoDAmix with widely used batch correction methods, including Harmony^17^, ComBat^16^, and Scanorama^18^. Harmony performs batch correction by iteratively adjusting low-dimensional embeddings to reduce batch-associated variation while preserving shared biological structure across datasets. Specifically, the method projects data into a reduced-dimensional space using principal component analysis (PCA) and applies an iterative clustering and correction procedure to align cells or samples across batches. ComBat is a statistical batch correction approach based on an empirical Bayes framework, which adjusts feature-wise means and variances by pooling information across samples to remove batch effects. Scanorama corrects batch effects by identifying mutual nearest neighbors across datasets to establish pairwise correspondences between batches and progressively aligns datasets into a shared low-dimensional space while preserving local neighborhood structure. All batch correction methods were evaluated using their default or recommended hyperparameter settings.

To more comprehensively assess multi-omics integration performance, MoDAmix was additionally compared with representative models designed specifically for multi-omics analysis, including MOFA+^30^, scMVAE^31^, GLUE^32^, and MultiVI^33^. MOFA+ is a factor analysis–based statistical framework that employs automatic relevance determination to infer a low-dimensional representation composed of latent factors capturing global sources of variability across multiple omics layers. In contrast, scMVAE, GLUE, and MultiVI are deep generative models primarily developed for single-cell multi-omics integration and are based on variational inference. scMVAE jointly models multiple omics modalities by learning modality-specific encoders together with a shared latent representation, capturing both shared and modality-specific sources of variation through probabilistic latent variables. GLUE employs omics-specific variational autoencoders to learn cell embeddings for each modality and leverages a guidance graph to encode cross-modality feature relationships, reconstructing omics data through inner products of learned embeddings. MultiVI learns modality-specific multivariate normal distributions using separate encoders for each omics layer and penalizes discrepancies between the resulting latent representations to encourage alignment. A unified latent space is obtained by averaging modality-specific embeddings, from which observations are generated using modality-specific decoders. All multi-omics integration methods were evaluated following the default or recommended settings provided in their original implementations.

## Supplementary Information

Supplementary Information.

## Data Availability

TCGA-LAML, TARGET-AML, TCGA-LGG, and CPTAC-3 datasets are available from GDC Data Portal (https://portal.gdc.cancer.gov/). Adult mouse brain dataset used in this study are available from GEO repository (https://www.ncbi.nlm.nih.gov/geo/) with the GEO accession of GSE130399 and GSE126074. Source codes of MoDAmix is publicly available at https://github.com/cbi-bioinfo/MoDAmix.
