# Supplementary material for: A unified framework for correcting batch effects and integrating multi-omics data
Source: Sci Rep. 2026 Mar 5;16:12341. doi: 10.1038/s41598-026-42355-9 (PMC13079841; doi:10.1038/s41598-026-42355-9)

Supplementary Material S1. Accuracy comparison of MoDAmix and widely-used batch correction methods based on 5-fold cross-validation on labeled source datasets across benchmark tasks.

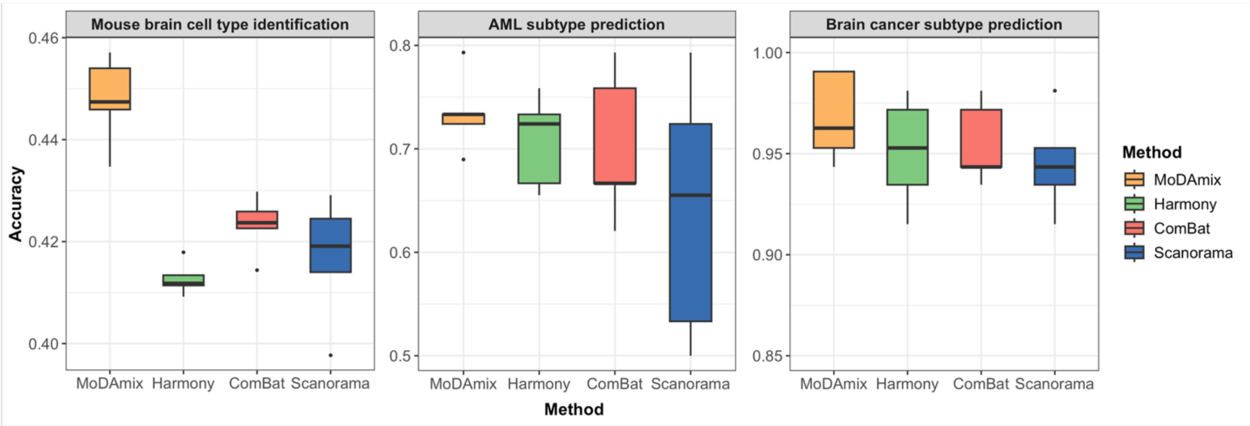

Supplementary Material S2. Average performance of MoDAmix and other batch correction methods based on 5-fold cross-validation on labeled source datasets across benchmark tasks.

Dataset	Accuracy				F1-score			
	MoDAmix	Harmony	ComBat	Scanorama	MoDAmix	Harmony	ComBat	Scanorama
Mouse brain	**0.448**	0.413	0.423	0.414	**0.440**	0.415	0.425	0.417
AML	**0.735**	0.708	0.701	0.641	**0.728**	0.706	0.695	0.631
Brain cancer	**0.966**	0.951	0.955	0.945	**0.967**	0.950	0.955	0.944

Supplementary Material S3. UMAP projections comparing uncorrected datasets and batch-corrected representations obtained from MoDAmix and other methods. For each dataset—(a–c) mouse brain, (d–f) acute myeloid leukemia (AML), and (g–i) brain cancer—the top panels show samples colored by batch, and the bottom panels show samples colored by predicted cell types or subtypes. (a, d, g) Uncorrected datasets before correction. (b, e, h) Multi-omics–integrated, batch-corrected features extracted using MoDAmix. (c, f, i) Batch-corrected representations from comparison methods, visualized based on either DNA accessibility (mouse brain) or DNA methylation (AML and Brain cancer) features.

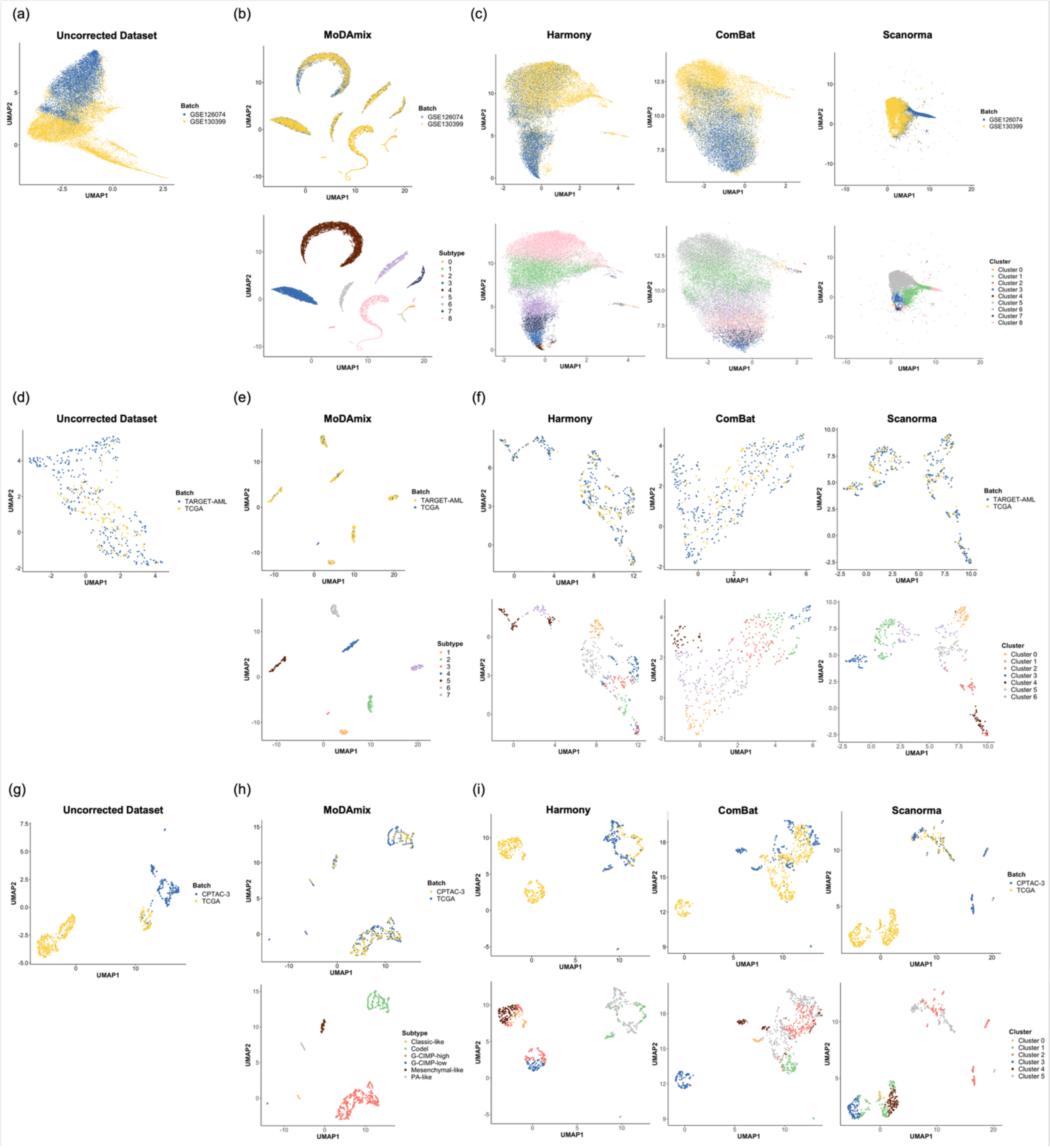

Supplement: Supplementary file 1 — Supplementary Information. [file 41598_2026_42355_MOESM1_ESM.pdf]
